# Insights Into the Mode of Action of the Anti-*Candida* Activity of 1,10-Phenanthroline and its Metal Chelates

**DOI:** 10.1155/MBD.2000.185

**Published:** 2000

**Authors:** Malachy McCann, Majella Geraghty, Michael Devereux, Denis O'Shea, James Mason, Luzveminda O'Sullivan

**Affiliations:** 1 Chemistry Department National University of Ireland Maynooth Maynooth, Co.m Kildare Ireland; 2 Dublin Institute of Technology Cathal Brugha Street Dublin Ireland; 3 Biochemistry Department Trinity College Dublin Ireland

## Abstract

Metal complexes of malonie acid (metal = Mn(II), Co(II), Ni(II), Cu(II), Zn(II), Ag(I)) were prepared and only the Ag(I) complex inhibited the growth of *Candida albicans*. Malonate complexes incorporating the chelating 1,10-phenanthroline (1,10-phen) ligand showed a range of activities: good (Mn(II), Cu(II), Ag(I)); moderate (Zn(II)); poor (Co(II), Ni(II)). Metal-free 1,10-phen and Ag(CH_3_CO_2_) were also highly active. The metal-free non-chelating ligands 1,7- phenanthroline and 4,7-phenanthroline were inactive and the Cu(II), Mn(II) and Zn(II) complexs of 1,7-phen displayed only marginal activity. Whereas the Cu(II) malonate/1,10-phen complex induces significant cellular oxidative stress the Zn(II) analogue does not.


### INSIGHTS INTO THE MODE OF ACTION OF THE ANTI-*CANDIDA* ACTIVITY OF 1,10-PHENANTHROLINE AND ITS METAL CHELATES

Malachy McCann*,[1] Majella Geraghty,[1] Michael Devereux,[2] Denis O'Shea,[2] James Mason[3] and Luzveminda O'Sullivan[3]

[1] Chemistry Department, National University of Ireland Maynooth, Maynooth, Co.m Kildare, Ireland <mmcann@may.ie>
[2] Dublin Institute of Technology, Cathal Brugha Street, Dublin, Ireland
[3] Biochemistry Department, Trinity College, Dublin, Ireland

**Abstract**

Metal complexes of malonic acid (metal = Mn(II), Co(II), Ni(II), Cu(II), Zn(II), Ag(I)) were prepared and only the Ag(I) complex inhibited the growth of *Candida albicans*. Malonate complexes incorporating the chelating 1,10-phenanthroline (1,10-phen) ligand showed a range of activities: good (Mn(II), Cu(II), Ag(I)); moderate (Zn(II)); poor (Co(II), Ni(II)). Metal-free 1,10-phen and $Ag(CH_3CO_2)$ were also highly active. The metal-free non-chelating ligands 1,7- phenanthroline and 4,7-phenanthroline were inactive and the Cu(II), Mn(II) and Zn(II) complexs of 1,7-phen displayed only marginal activity. Whereas the Cu(II) malonate/1,10-phen complex induces significant cellular oxidative stress the Zn(II) analogue does not.

*Keywords*: 1,10-phenanthroline; metal complexes; *Candida albicans*; oxidative stress.

### Introduction

1,10-Phenanthroline (1,10-phen), 2,2′-bipyridine (2,2′-bipy) (Fig. 1) and their substituted derivatives, both in the metal-free state and as ligands coordinated to transition metals, disturb the functioning of a wide variety of biological systems.[1] When the metal-free N,N-chelating bases are found to be bioactive it is usually assumed that the sequestering of trace metals is involved, and that the resulting metal complexes are the actual active species.[2,3]

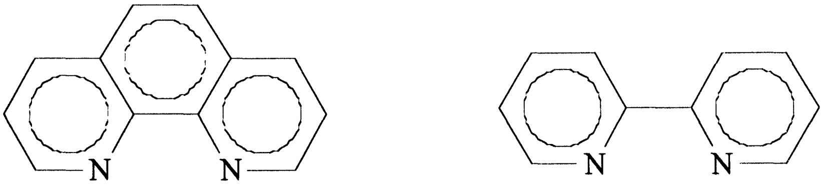

(a) (b)

**Figure 1.** (a) 1,10-Phenanthroline (1,10-phen); (b) 2,2′-bipyridine (2,2′-bipy)

Administering the metal-free bases is known to affect succinic oxidase,[4] triphosphopyridine nucleotide nitrate reductase,[5] zymase[6] and the reduction of fumerate and malate by molecular hydrogen in the presence of washed suspensions of *Escherichia coli.*[7] The action of liver and intestine phosphomonoesterases on β-glycerophosphate and of intestine pyrophosphotase on pyrophosphate is also considerably accelerated by solutions of the bases.[8,9] Whilst 1,10-phen slows cysteine oxidation[10] 2,2′-bipy and the Fe(II) complex $[Fe(2,2'\text{-bipy})_3]^{2+}$ activate cathepsin[11] and also catalyse the autoxidation of linoleic acid.[12] Whereas the oxygen uptake of green leaves[13] and the endogeneous respiration of anabaema[14] are inhibited by the bases, both 1,10-phen and 2,2′-bipy accelerate the oxygen consumption of homogenised brain brei.[10] Highly potent anthelmintic action has been observed with both bases.[15] Furthermore, a solution of 1,10-phen suppresses the chemotactic power of "guinea pig" leucocytes without destroying the cells.[16]

The *in vitro* antibacterial action of 1,10-phen has been demonstrated on several species of bacteria.[3,17-21] Whereas phenanthroline metal complexes can be bacteriostatic[3] and bacteriocidal[20] towards many Gram-positive bacteria they are relatively ineffective against Gram-negative organisms. Similar reactivity trends against Gram-positive and Gram-negative bacteria were found for Cu(II) and Fe(II/III) complexes of oxine.[21]

Whereas *m*- and *p*-substituted phenanthrolines were less effective than 1,10-phen at preventing fungal growth, 2,9-dimethyl-1,10-phenanthroline (dmphen) was the most potent inhibitor.[22] Recent *in vitro* studies have shown that although 2,2′-bipy and its metal complexes did not suppress the growth of clinical isolates of *Candida* species dilute aqueous solutions of 1,10-phen and its Cu(II) and Mn(II) complexes were extremely toxic to the cells.[23,24]

Metal complexes of phenanthroline and bipyridine are toxic to mice and rats[25] and to frogs and rabbits[26], and their action on enzymes[10,25,27] and on neuromuscular transmission[28] has been demonstrated. $[Fe(2,2'\text{-bipy})_3]^{2+}$ is excreted unchanged through the kidneys of frogs and rabbits,[26,29-32] and experiments using radiolabelled $Ru^{106}$ have revealed that $[Ru(1,10\text{-phen})_3]^{2+}$ is not metabolised in rats and mice.[25] Very small amounts were lethal when introduced intraperitoneally, intravenously or subcutaneously, but even large oral doses did not cause death. Absorption through the intestine was slow (this has also been observed on humans[33]) and, owing to rapid elimination through the kidney, it was not possible to build up the lethal concentration in the blood. The symptoms caused by the complexes, i.e., torpor, tremor, paralysis of the limbs, chronic convulsions, an unaffected heart, and death due to respiratory failure, indicated an attack on the central nervous system. These conclusions were supported by findings that the complexes are potent inhibitors of acetylcholinesterase and that they have a curare-like action on neuromuscular transmission. Normal activity was restored by washing out the complexes.

The *in vitro* antitumour activities of Cu(II) complexes of 3,5-disubstituted salicylates increased by an order of magnitude by incorporation of a phenanthroline ligand into the complex.[34] These ternary complexes had cytotoxicities comparable to that of the anticancer drug cisplatin, *cis*-$[PtCl_2(NH_3)_2]$. However, although $[Cu(dmphen)(Hdips)_2]$ (Hdips = 3,5-diisopropylsalicylate) had potent *in vitro* cytotoxicity it was inactive when tested on mice bearing tumour, suggesting that it is destroyed by interaction with biofluids and is not stable enough to reach the tumour cells *in vivo*.

*In vitro* experiments revealed that metal complexes of 3,5,6,8- or 3,4,7,8-tetramethyl-1,10-phen (metal = Ni(II), Fe(II), Co(II), Cu(II), Zn(II), Cd(II), Mn(II), Ru(II)), the metal-free hydrochlorides and quaternary salts of the bases were bactericidal to veterinary samples of *Erysipelothrix rhusiopathiae* and *Fusiformis nodosus*.[35] Resistant variants of *E. rhusiopathiae* were not produced by repeated subculture in the presence of $[Cu(3,4,7,8\text{-tetramethyl-}1,10\text{-phen})_2]X_2$ (X = benzoate or acetate). Likewise, *Staphylococcus aureus, Mycobacterium tuberculosis, E. coli, Candida albicans* and *Trichophyton mentagrophytes* develop little resistance to this type of complex.[3,36] On the basis of these experiments it was suggested that the complexes could possibly be used to treat erysipelas in pigs, and may also be beneficial in the treatment of other topical infection of bacteria, mycotic or even viral origin. Furthermore, the latter Cu(II) complex was shown to be at least as effective as solutions of formalin and dioxyethyl laural ammonium chloride in the treatment of foot-rot in sheep. While useful clinically as topical antimicrobials, a selection of phenanthroline complexes, when administered parenterally, were found to be chemptherapeutically ineffective against experimental infection of mice with *S. aureus, Streptococcus pneumoniae* and *M. tuberculosis*.[3] Also, the complexes did not decrease ulcer growth rate in guinea-pigs infected with *M. tuberculosis*.

Extensive microbiological and pharmacological investigations with phenanthrolines and related chelates[3,36] led to clinical studies with the highly stable Ni(II) and Fe(II) complexes of 3,4,7,8-tetramethyl-1,10-phen. These complexes were known to have a wide spectrum of antimicrobial actions and to produce negligible toxicity to skin, subcutaneous tissues and mucous membranes.[36] Preliminary studies had shown that the complexes were effective in controlling infections due to *S. aureus, C. albicans* and selected dermatophytes found in hospital casualty, dermatology, ear, nose and throat and gynaecology departments and in surgical wards. The Ni(II) complex was equally as effective as hexachlorophene, when used as an immediate preoperative skin preparation, in decreasing the incidence of postoperative staphylococcal wound infection in elective abdominal surgery. Further trials established that the same complex was again as effective as hexachlorophene in the prophylaxis of staphylococcal infection in the newly born and also in patients undergoing elective obstetric or gynaecological surgery.[37] The complex was also beneficial in controlling secondary infection in adolescents with acne vulgaris of long standing. The Ni(II) and the Cu(II) complexes provided rapid relief of symptons in patients with either chronic monilial infection of the nail or trichomonal vaginitis, attributable to *C. albicans* and *Trichophyton vaginalis*, respectively. Furthermore, no toxic manifestations were observed with either substance.

Mn(II) complexes of 3,4,7,8-tetramethyl-1,10-phen were used topically to treat patients suffering from a variety of skin conditions, many of whom had chronic dermatological infections due to dermatophytes (e.g. *Malassezia furfur*, *Trichophyton rubrum*) or *Candida* species.[38] The complexes produced a significant decrease in microbial infection in approximately 50% of cases, with infection due to Gram-positive bacteria generally responding much more rapidly and readily to treatment than that due to Gram-negative bacteria. The complexes did not produce noteworthy irritation or sensitization of the underlying dermatosis or dermatomycosis and, furthermore, significant microbial resistance did not develop.

### Materials and Methods

All chemicals were reagent grade and used without further purification. Reactions involving silver salts were carried out in the dark. Infrared spectra were recorded as KBr discs in the region 4000-400 $cm^{-1}$ on a Nicolet Impact 400D FT-IR Spectrometer. The spectra of the metal carboxylate complexes contained prominent $\nu_{asym}OCO$ and $\nu_{sym}OCO$ stretching bands at *ca.* 1600 $cm^{-1}$ and *ca.* 1400 $cm^{-1}$, respectively.[39] Additional characteristic bands were exhibited by the 1,10-phen ligand (*ca.* 855 $cm^{-1}$ and *ca.* 743 $cm^{-1}$). Elemental analysis were carried out by the Microanalytical Laboratory, National University of Ireland, Cork, Ireland. Biological studies were carried out in the Labsystems iEMS reader MF.

**[Mn(mal)].$H_2O$.** To a solution of malonic acid {$HO_2CCH_2CO_2H$, $malH_2$} (10.0 g, 96 mmol) in ethanol (40 $cm^3$) was added $Mn(CH_3CO_2)_2.4H_2O$ (2.6 g, 10.6 mmol). The resulting solution was allowed to reflux for 0.5h. The resulting white solid which deposited was filtered off, washed with water and ethanol, and then air-dried.

**[Co(mal)]$malH_2.2H_2O$, [Cu(mal)].$H_2O$ and [Zn(mal)]$malH_2.2H_2O$** were made in a similar manner to [Mn(mal)].$H_2O$ using $Co(CH_3CO_2)_2.4H_2O$, $[Cu_2(CH_3CO_2)_4(H_2O)_2]$ and $Zn(CH_3CO_2)_2.2H_2O$, respectively.

**$[Ni(mal)(H_2O)_2]$ and $[Ag_2(mal)]$ (1)** were prepared from $Ni(CH_3CO_2)_2.4H_2O$ and $Ag(CH_3CO_2)$ and using water as the reaction solvent.

**$[Mn(1,10\text{-}phen)_2(mal)].2H_2O$ (2).** To a solution of 1,10-phenanthroline monohydrate (2.20 g, 11.1 mmol) in ethanol (40 $cm^3$) was added [Mn(mal)].$H_2O$ (0.75 g, 2.1 mmol) and the yellow solution was refluxed for 0.5 h. The precipitated yellow solid was filtered off, washed with water and ethanol, and then air-dried.

**$[Co(1,10\text{-}phen)_3]mal.H_2O$ (3), $[Cu(1,10\text{-}phen)_2(mal)].2H_2O$ (4), $[Ni(1,10\text{-}phen)_2(mal)].2H_2O$ (5), $[Zn(1,10\text{-}phen)_2(mal)].2H_2O$ (6)** and **$[Ag_2(1,10\text{-}phen)_3(mal)].2H_2O$ (7)** were prepared in a similar manner to **2**.

**$[Mn(1,10\text{-}phen)_2](CH_3CO_2)_2.4H_2O$ (8).** To a solution of 1,10-phenanthroline hydrate (1.78 g, 8.98 mmol) in ethanol (40 $cm^3$) was added $Mn(CH_3CO_2)_2.4H_2O$ (1.0 g, 4.08 mmol) and the resulting yellow solution was refluxed for 1 h. The solution was then concentrated by rotary evaporation to encourage precipitation of the yellow product. The solid was washed with a small volume of water and ethanol, and then air-dried.

**$[Cu(1,10\text{-}phen)(CH_3CO_2)_2].H_2O$ (9)** and **$[Zn(1,10\text{-}phen)(CH_3CO_2)_2].2H_2O$ (10)** were made in a similar manner to **8** using $[Cu_2(CH_3CO_2)_4(H_2O)_2]$ and $Zn(CH_3CO_2)_2.2H_2O$, respectively.

**$[Mn(1,10\text{-}phen)_2Cl_2]$ (11).** To a solution of 1,10-phenanthroline hydrate (1.10 g, 5.55 mmol) in ethanol (40 $cm^3$) was added $MnCl_2.4H_2O$ (0.50 g, 2.53 mmol) and the resulting yellow solution refluxed for 1 h. Upon cooling the resulting yellow crystals which deposited were filtered off, washed with a small volume of ethanol and water, and then air-dried.

**$[Cu(1,10\text{-}phen)Cl_2].H_2O$ (12)** and **$[Zn(1,10\text{-}phen)Cl_2].H_2O$ (13)** were made in a similar manner to **11** using $CuCl_2.2H_2O$ and $ZnCl_2$, respectively.

**$[Mn(1,7\text{-}phen)_2(CH_3CO_2)_2]$ (14).** To a solution of 1,7-phenanthroline (1,7-phen) (0.5 g, 2.8 mmol) in ethanol (40 $cm^3$) was added $Mn(CH_3CO_2)_2.4H_2O$ (0.15 g , 0.61 mmol) and the resulting yellow solution was refluxed for 1 h. The solution was then evaporated down to 5 $cm^3$ by rotary evaporation, and then acetone (20 $cm^3$) was added. The resulting solution was then evaporated to 3 $cm^3$ and diethyl ether (7 $cm^3$) added. This solution was allowed to evaporate in air over a period of 24 h and the resulting brown crystals were filtered off, washed with diethyl ether and ethanol, and then air-dried.

**$[Cu(1,7\text{-}phen)(CH_3CO_2)_2].2H_2O$ (15)** and **$[Zn(1,7\text{-}phen)(CH_3CO_2)_2].H_2O$ (16)** were made in a similar manner to **14** using $[Cu_2(CH_3CO_2)_4(H_2O)_2]$ and $Zn(CH_3CO_2)_2.2H_2O$, respectively.

Satisfactory microanalysis were obtained for all complexes and complexes **(1)-(16)** were water-soluble.

### Anti-*Candida* susceptibility testing

*C. albicans* (one clinical isolate) was obtained from St. James's Hospital, Dublin, Ireland. The isolates were stored on Sabouraud dextrose agar (SDA) plates at 4 °C. Solutions of the water-soluble test complexes **1-16** were prepared by dissolving the complex (0.02 g) in distilled water (100 $cm^3$) to yield a stock solution with a concentration of 200 $\mu g\ cm^{-3}$. Doubling dilutions of this stock solution were made to yield a series of test solutions ranging in concentration from 200 - 0.195 $\mu g\ cm^{-3}$.

RPMI-1640 broth medium (Sigma R 7755) was used for the anti-*Candida* susceptibility testing. The medium (1 $dm^3$) was supplemented with L-glutamine (0.3 g) and morpholinepropanesulfonic acid (MOPS) (34.6 g) and was then adjusted to pH 7.0 using sterile NaOH (0.2 M). The broth microdilution reference method was used.[40] Prior to testing, yeast cells were grown on Sabouraud dextrose agar (SDA) at 37 °C for 24 h. Cell suspensions were prepared in sterile phosphate buffered saline (5 $cm^3$) to a density of 0.5 McFarland standard. A 1:100 dilution of these cell suspensions were made in RPMI-1640 medium so that the cell concentration of the final inoculum was $3.5 \times 10^4$ - $5.0 \times 10^5$ cells $cm^{-3}$. The prepared cell suspension (90 µl) was dispensed into sterile microtitre test plates and to this was added the complex test solution (10 µl) to yield working solutions of the test complexes of concentration 20 - 0.0195 µg $cm^{-3}$. Minimum inhibitory concentration (MIC) determination on selected complexes was carried out for 24 h at 37 °C with continuous shaking. Each complex was assessed in triplicate and three independent experiments were performed. The resulting nine data points were statistically analysed using ANOVA one-way analysis of variance followed by Tukey's family error rate.

**Oxidative stress studies**

Yeast cells were grown on SDA at 37 °C for 24 h. Cell suspensions were then prepared in sterile phosphate buffered saline (PBS, 5 $cm^3$) to a density of 0.5 McFarland standard. A 1:100 dilution of these cell suspensions was made in RPMI-1640 medium (30 $cm^3$) to give a cell concentration in the final inoculum of $3.5 \times 10^4$ - $5.0 \times 10^5$ cells $cm^{-3}$. The prepared cell suspension was dispensed into sterile Erylenmeyer flasks and the flasks then incubated in a shaking water bath for 24 h at 37 °C with continuous shaking. Cell growth was assessed using an improved Neubauer haemocytometer chamber.

Cells growing in the mid exponential phase (9 h) were collected by centrifugation (3,000 rpm for 10 min), washed twice with sterile PBS and suspended in PBS (9 $cm^3$). To this was added 1 $cm^3$ of the stock complex solution (complex **4** or **6**; 0.2 g in 100 $cm^3$ of water) to yield working solutions of the test complexes of concentration 200 µg $cm^{-3}$. The cell suspension was then incubated at 37 °C for 1 h with gentle shaking. Cells were collected by centrifugation (3,000 rpm for 10 min), washed three times with PBS and then suspended in ice-cold PBS (5 $cm^3$).

Cell viability studies were conducted by diluting the cells 1,000-fold with sterile distilled water. Serial dilutions were performed so that approximately 100 cells were plated onto individual SDA plates. The plates were incubated at 37 °C for 24 h and the number of colonies counted. Cell viability is expressed as % cells surviving after treatment with drug compared with an untreated control.

Spheroplasts were prepared as follows. Glass beads were added to test and control cell suspensions and the cells disrupted using a whirlimixer (30 cycles each of 2 min duration). In between cycles cells were placed on ice. Cell wall removal was observed by light microscopy. The spheroplasts were separated from the glass beads by aspiration and collected by centrifugation (3,000 rpm for 15 min). The resulting spheroplast pellet was resuspended in PBS (10 $cm^3$) and samples of this were used separately for protein estimation, lipid peroxidation and glutathione assays.

Protein estimation on the spheroplasts was carried out using the Protein Assay Kit, Sigma P 5656.

*Lipid peroxidation.* To ascertain if there were differences in lipid peroxidation in the absence and in the presence of a metal complex the formation of lipid peroxides was indirectly determined spectrophotometrically by a modification of the published method.[41] Malonaldehyde (MDA) is a secondary product of lipid peroxidation. Thiobarbituric acid reacts with linoleic acid hydroperoxide (a product of lipid peroxidation) in a heated reaction to form MDA and other compounds. Since MDA is not the only product of peroxidation the assay is not a direct measurement of MDA, although this is used as a standard. A pink colour is formed by this reaction ($\lambda$ = 540 nm) and an increased absorbance value is indicative of more extensive lipid peroxidation and oxidative damage.

The reaction mixture comprised the spheroplast suspension (0.2 $cm^3$, 220 µg protein), aqueous sodium dodecyl sulphate (8.1% w/v, 0.2 $cm^3$), aqueous acetic acid (20% v/v, adjusted to pH 3.5 with NaOH, 1.5 $cm^3$), aqueous 2-thiobarbituric acid (0.8% w/v, 1.5 $cm^3$). The mixture was finally made up to 4 $cm^3$ with deionised water and then heated at 95 °C for 1 h. After cooling to room temperature the mixture was filtered (0.45 µm membrane filter) and its absorbance measured.

*Glutathione assay.* Total gluthathione {reduced glutathione (GSH) plus oxidised glutathione (GSSG)} was determined using a minor modification of the published method.[42] Prior to testing the spheroplast suspension (5 $cm^3$) was centrifuged and the resulting pellet was quickly mixed with 5-sulphosalicylic acid (10% w/v, 5 $cm^3$). The sample was centrifuged and the supernatant retained. To a reaction mixture comprising β-nicotinamide adenine di-nucleotide phosphate (reduced form, NADPH, 0.3 mM, 700 μl), 5,5'-dithio-bis(2-nitrobenzoic acid) (DTNB) (6.0 mM, 100 μl) and triethanolamine (20 μl) was added the above supernatant (200 μl) and the mixture then equilibrated to 37 °C. To the solution was added glutathione reductase (50 units $cm^{-3}$, 10 μl) and total glutathione content (GSH + GSSG) was estimated spectrophotometrically (_ = 412 nm) by comparison to the rate of reduction with known amounts of DTNB.

GSH content alone was determined using the same procedure as that described above for total glutathione with the exception that 2-vinyl-pyridine (10 μl) was added prior to the addition of the glutathione reductase.

**Results and Discussion**

At a concentration of 20 μg $cm^{-3}$ malonic acid has previously been reported to have negligible inhibitory effect on *C. albicans*.[23] In addition, the present study has shown that the simple metal malonates (metal = Mn(II), Co(II), Ni(II), Cu(II), Zn(II)) and $Ni(CH_3CO_2)_2.4H_2O$ also have negligible activity against the yeast.

MIC values were determined for metal-free 1,10-phen, $Ag(CH_3CO_2)$, $[Ag_2(mal)]$ (**1**) and the metal-1,10-phen complexes **2-7** on the growth of *C. albicans* (Table 1). Values for metal-free 1,10-phen, $Ag(CH_3CO_2)$, and the Mn(II), Cu(II) and Ag(I) 1,10-phen complexes (**2**, **4** and **7**, respectively) all fell within the range 1.25-2.5 μg $cm^{-3}$. The fact that the activity of metal-free 1,10-phen and its Cu(II) complex **4** are quite similar contrasts somewhat with the observations of Seydel *et al*[43] who found that addition of Cu(II) ions to derivatised 1,10-phenanthroline ligands led to a decrease in the anti-*Candida* activities of the metal-free ligands.

MIC values increased progressively for the Zn(II) 1,10-phen complex **6** and the Ag(I) malonate **1** (>5 μg $cm^{-3}$), and substantially higher MIC values (>20 μg $cm^{-3}$) were found for the Co(II) and Ni(II) 1,10-phen complexes (**3** and **5**, respectively). Results for the three Ag(I) complexes indicate that it is the $Ag^+$ ion itself that is the active species and that neither the presence of 1,10-phen or the nature of the carboxylate anion are contributory factors in determining activity.

**Table 1.** MIC of 1,10-phen, metal-1,10-phen complexes and selected starting materials on *C. albicans.*

| Complex | μg $cm^{-3}$ |
|---|---|
| 1,10-phen | 1.25-2.5 |
| $[Mn(1,10\text{-phen})_2](mal)_2.2H_2O$ (2) | 1.25-2.5 |
| $[Co(1,10\text{-phen})_3](mal)_2.H_2O$ (3) | >20 |
| $[Cu(1,10\text{-phen})(mal)_2].2H_2O$ (4) | 1.25-2.5 |
| $[Ni(1,10\text{-phen})_2](mal)_2.2H_2O$ (5) | >20 |
| $[Zn(1,10\text{-phen})(mal)_2].2H_2O$ (6) | >5 |
| $Ag(CH_3CO_2)$ | 1.25-2.5 |
| $[Ag_2(mal)]$ (1) | >5 |
| $[Ag_2(1,10\text{-phen})_3(mal)].2H_2O$ (7) | 1.25-2.5 |

Compounds were tested within a concentration range of 20 - 0.0195 μg $cm^{-3}$ of aqueous RPMI medium.

It is significant that $Ag(CH_3CO_2)$ is the only complex not to contain 1,10-phenanthroline but to exhibit activity similar to that of metal-free 1,10-phen and other metal complexes containing the 1,10-phen ligand. Previous studies have shown that the MIC values of simple metal acetates (metal = Mn(II), Cu(II) and Co(II))[44] is >20 μg $cm^{-3}$. In addition, by studying the anti-*Candida* activity of a series of complexes (concentration = 20 μg $cm^{-3}$) of general formula $[M(1,10\text{-phen})_n]X_y$ (M = Mn(II), Cu(II), Zn(II); X = malonate, acetate, chloride) it was established that the nature of the counter anion X does not influence the inhibitory effect (Table 2).

In contrast to the performance of the highly active metal-free 1,10-phen ligand metal-free 1,7- and 4,7-phenanthroline (Fig. 2) were both inactive and, furthermore, Cu(II), Mn(II) and Zn(II) complexes of 1,7-phen displayed only marginal activity (Table 2).

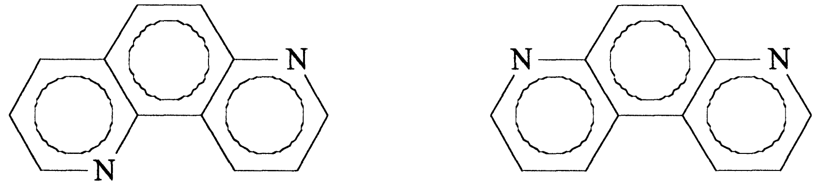

Figure 2. (a) 1,7-Phenanthroline (1,7-phen); (b) 4,7-phenanthroline (4,7-phen).

Although all of the phenanthroline isomers can coordinate to metal centres 1,10-phen is the only ligand capable of actually chelating the metal and forming an extremely stable metal-phen entity in solution. These observations would appear to substantiate the hypothesis that the bioactivity of N,N-chelating bases is attributed to their ability to sequester specific transition metals and that it is the resulting metal chelate complex that are the active species.

**Table 2.** Anti-*Candida* activity of [M(1,10-phen)$_n$]X (M = Mn(II), Cu(II), Zn(II); X = malonate, acetate, chloride), [M(1,7-phen)$_n$]acetate (M = Mn(II), Cu(II), Zn(II) and metal free phen ligands.

| Complex | % Cell growth |
|---|---|
| [Mn(1,10-phen)$_2$](mal)$_2$.2$H_2O$ (2) | 0 |
| [Cu(1,10-phen)(mal)$_2$].2$H_2O$ (4) | 0 |
| [Zn(1,10-phen)(mal)$_2$].2$H_2O$ (6) | 89 ± 9 |
| [Mn(1,10-phen)$_2$]($CH_3CO_2$)$_2$.4$H_2O$ (8) | 2 ± 0.1 |
| Cu(1,10-phen)($CH_3CO_2$)$_2$].$H_2O$ (9) | 8 ± 2 |
| [Zn(1,10-phen)($CH_3CO_2$)$_2$].2$H_2O$ (10) | 80 ± 9 |
| [Mn(1,10-phen)$_2Cl_2$] (11) | 3 ± 0.001 |
| [Cu(1,10-phen)$Cl_2$].$H_2O$ (12) | 5 ± 2 |
| [Zn(1,10-phen)$Cl_2$].$H_2O$ (13) | 90 ± 4 |
| [Mn(1,7-phen)$_2$($CH_3CO_2$)$_2$] (14) | 83 ± 5 |
| [Cu(1,7-phen)($CH_3CO_2$)$_2$].2$H_2O$ (15) | 89 ± 9 |
| [Zn(1,7-phen)($CH_3CO_2$)$_2$].$H_2O$ (16) | 97 ± 12 |
| 1,7-phen | 99 ± 9 |
| 4,7-phen | 93 ± 13 |
| 1,10-phen | 0 |

Complexes were tested at a concentration of 20 μg $cm^{-3}$ of aqueous RPMI medium. Yeast cells were grown for 24 h at 37 °C. Results are presented as % cell growth and the effectiveness of the compounds are compared to the growth of the control (no added complex).

The kinetic growth profile of the yeast when it was exposed to sub-inhibitory concentration of [Mn(1,10-phen)$_2$(mal)].2$H_2O$ (**2**) (0.156 μg $cm^{-3}$) is shown in Fig. 3. Control cells and cells treated with complex **2** enter the early exponential phase at the same time (4 h). However, after this stage growth of the Mn-treated cells is significantly reduced (doubling time approximately 600 min) when compared to the control (120 min). This result suggests that the cells have to metabolically active before the administered metal complexes take effect.

The MIC studies revealed two distinct groups of complexes exhibiting extremes of growth inhibition. The Mn(II), Cu(II) and Ag(I) complexes (**2**, **4** and **7**, respectively) were highly toxic, whereas the Co(II), Ni(II) and Zn(II) complexes (**3**, **5** and **6**, respectively) were ineffective. On the basis of these results a representative complex was selected from each group ([Cu(1,10-phen)$_2$(mal)].2$H_2O$ (**4**) and [Zn(1,10-phen)$_2$(mal)].2$H_2O$ (**6**)) for oxidative stress studies. Cells were grown to mid-exponential phase and separate samples of these cells (each batch containing approximately 2 x $10^{10}$ cells) were treated with **4** and **6** (200 _g $cm^{-3}$) for 1 h at 37 °C. The viability of cells treated with **4** (9 ± 2%) and **6** (80 ± 9%) highlights the pronounced toxicity of the copper complex (Table 3). GSH is known to be an important antioxidant molecule in yeast cells.[45,46] GSH:GSSG ratios determined for the control (17:1), [Zn(1,10-phen)$_2$(mal)].2$H_2O$ (**6**) (22:1) and [Cu(1,10-phen)$_2$(mal)].2$H_2O$ (**4**) (5:1) clearly demonstrates that the copper complex induces significant cellular oxidative stress. Furthermore, whereas cells treated with the Zn(II) complex **6** showed no elevation in the level of lipid peroxides (compared to the control) those exposed to the Cu(II) complex **4** had a 7-fold increase. In relation to this result it is known that, as well as targeting ergosterol in the fungal cell membrane and causing pore formation, the polyene antifungal amphotericin B also promotes peroxidation of membrane lipids in *Candida* cells.[47] *C. albicans* when exposed to a low dose of gamma-radiation is also known to undergo lipid peroxidation.[41]

**Figure 3.** Kinetic growth curve of control cells and cells treated with [Mn(1,10-Phen)$_2$](mal)$_2$.4$H_2O$ (**2**).

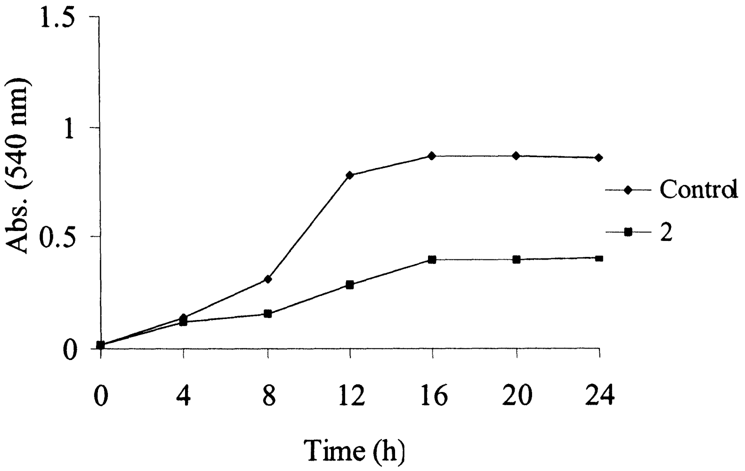

The kinetics of a sub-inhibitory concentration (0.156 μg $cm^{-3}$) of complex **2** was followed spectrophotometrically (abs. 540 nm). The cells were grown in RPMI aqueous medium and readings were taken every 4 h over a 24 h period. The results presented are a representative of a total of three independent experiments.

**Table 3**. Cell viability, protein estimation, lipid peroxide level and GSH:GSSG.

| | Viability [a] (%) | Protein (μg/ml) | Lipid peroxides [b] | GSH:GSSG [c] |
|---|---|---|---|---|
| Control | 100 | 1173 ± 12 | 1:1 | 17:1 |
| Complex (4) | 9 ± 2 | 1196 ± 22 | 7:1 | 5:1 |
| Complex (6) | 80 ± 9 | 1104 ± 29 | 1:1 | 22:1 |

[a] Viability is expressed as the % cells surviving (mean ± se (*n* = 9)) after treatment with drug compared to an untreated control. [b] Lipid peroxide formation was measured at an absorbance at 540 nm. The level of lipid peroxidation was obtained by comparing the absorption of the test drugs compared to the absorption of the control. The results are expressed as the mean of three independent experiments. [c] The ratio of reduced glutathione (GSH) and oxidized glutathione (GSSG) was calculated from the absorbance at 412 nm using the rate of reduction of DTNB as an external standard, and expressed as mean of three independent experiments.

In conclusion, it seems possible that the heightened anti-*Candida* activity of the bis(1,10-phen) Mn(II) and Cu(II) complexes, in contrast to that of the Ni(II) and Zn(II) analogous, may be attributed to the fact that the metal centers in the former can have variable oxidation states and are thus potential Fenton reagents. Indeed, the ability of redox-active phenanthroline and bipyridine metal complexes to catalyze the oxidative modification of proteins, nucleic acids and lipids has previously been attributed to the interactions of the substrate with $H_2O_2$ and the metal; i.e. to simple Fenton-type chemistry.[48] The inability of the metal centers in the present Ni(II) and Zn(II) 1,10-phen complexes to redox-cycle would render them incapable of assuming the role of a Fenton reagent. Although the tris(1,10-phen)Co(II) complex (**3**) would be expected to have a readily accessible and reversible Co(III) oxidation state the metal is coordinatively saturated with respect to 1,10-phen and the complex is thus likely to be extremely kinetically inert.

The consistently high activity displayed by the three different Ag(I) complexes, $Ag(CH_3CO_2)$, $[Ag_2(mal)]$ (**1**) and $[Ag_2(1,10\text{-phen})_3(mal)].2H_2O$ (**7**), indicates that in these instances it is the metal center and not the ligands that dictates complex performance. As Ag(I) is renowned for its ability to bind strongly to S-donor ligands[25] it is likely that the silver complexes will have a high affinity for the sulphur atoms of thiol proteins and may, ultimately, induce protein inactivation.

**References**

1. W. W. Brandt, F. P. Dwyer and E. C. Gyarfas, *Chem. Rev.*, 1954, **54**, 959.
2. R. A. MacLeod, *J. Biol. Chem.*, 1952, **197**, 751.
3. F. P. Dwyer, I. K. Reid, A. Shulman, G. M. Laycock and S. Dixon, *Aust. J. Exp. Biol. Med. Sci.*, 1969, **47**, 203.
4. S. R. Ames, H. J. Ziegenhagen and C. A. Elvehjem, *J. Biol. Chem.*, 1946, **165**, 81.
5. A. Nason and H. J. Evans, *J. Biol. Chem.*, 1953, **202**, 655.
6. F. Zuckerkandl and L. Messiner-Klebermass, *Biochem. Z.*, 1933, **261**, 55.
7. J. Lascelles and J. L. Still, *Proc. Linnean Soc.*, N. S. Wales, 1947, **72**, 49; *Chem. Abs.*, 1947, **41**, 7435.
8. C. Dumazert, M. Levy and I. Marszak, *Compt. Rend. Soc. Biol.*, 1945, **139**, 99.
9. C. Dumazert, M. Levy and I. Marszak, *Compt. Rend. Soc. Biol.*, 1945, **139**, 219.
10. F. Panimon, M. K. Horwitt and R. W. Gerard, *J. Cellular Comp. Physiol.*, 1941, **17**, 17.
11. L. Michaelis and K. G. Stern, *Biochem. Z.*, 1931, **240**, 192.
12. W. Franke, *Ann.*, 1932, **498**, 129.
13. G. Stenlid, *Plantarum*, 1949, **2**, 61.
14. G. C. Webster and A. W. Frenkel, *Plant Physiol.*, 1953, **28**, 63.
15. E. Baldwin, *Brit. J. Pharmacol.*, 1948, **3**, 91.
16. J. Lebrun and A. Delauney, *Ann. Inst. Pasteur*, 1951, **80**, 524.
17. G. Turian, *Schweiz. Z. allgem. Pathol. U. Bakteriol.*, 1951, **14**, 338. (see *Chem. Abs.*, 1951, **45**, 9124.
18. R. E. Feeney, I. M. Petersen and H. Sahinkaya, *J. Bact.*, 1957, **73**, 284.
19. M. L. McNaught and E. C. Owen, *Intern. Congr. Biochem., Abstr. of Communs., 1st Congr., Cambridge, England*, 1949, 304.
20. H. M. Butler, A. Hurse, E. Thursky and A. Shulman, *Aust. J. Exp. Biol. Med. Sci.*, 1969, **47**, 541.
21. *Metals and Micro-organisms* by M. N. Hughes and R. K. Poole, Chapman and Hall, London, 1989, 299.
22. F. Blank, *Nature*, 1951, **168**, 516.
23. M. Geraghty, J. F. Cronin, M. Devereux and M. McCann, *BioMetals*, 2000, **13**, 1.
24. M. Geraghty, J. F. Cronin, M. Devereux and M. McCann, *Polyhedron*, 1998, **18**, 2931.
25. F. P. Dwyer, E. C. Gyarfas, W. P. Rogers and J. H. Koch, *Nature*, 1952, **170b**, 190.
26. E. Beccari, *Boll. Soc. Ital. Biol. Sper.*, 1938, **13**, 6.
27. L. Michaelis and K. G. Stern, *Biochem. Z.*, 1931, **240**, 192.
28. E. Beccari, *Arch. Sci. Physiol.*, 1949, **3**, 611.
29. E. Beccari, *Boll. Soc. Ital. Biol. Sper.*, 1938, **13**, 8.
30. E. Beccari, *Boll. Soc. Ital. Biol. Sper.*, 1941, **16**, 214.
31. E. Beccari, *Boll. Soc. Ital. Biol. Sper.*, 1941, **16**, 216.
32. E. Beccari, *Arch. Sci. Biol., (Italy)*, 1941, **27**, 204.
33. J. Rechenberger and C. Pollack, *Z. Physiol.Chem.*, 1944, **281**, 186.
34. J. D. Ranford, P. J. Sadler and D. A. Tocher, *J. Chem. Soc., Dalton Trans.*, 1993, 3393.
35. G. Cade, M. Cohen and A. Shulman, *Aust. Vet. J.*, 1970, **46**, 387.
36. A. Shulman and F. P. Dwyer in *Chelating Agents and Metal Chelates*, ed. F. P. Dwyer and D. P. Mellor, Academic Press, New York, 1931, 383.
37. H. M. Butler, J. C. Laver, A. Shulman and R. D. Wright, *Med. J. Aust.*, 1970, **2**, 309.
38. G. Cade, K. H. Shankley, A. Shulman, R. D. Wright, I. O. Stahle, C. B. Maggibbon and E. Lew-Sang, *Med. J. Aust.*, 1970, **2**, 304.

39. K. Nakamoto, *Infrared and Raman Spectra of Inorganic and Coordination Compounds*, 5th edn., Part B, Wiley, New York, 1997.
40. NCCLS (National Committee for Clinical Laboratory (1979). Reference method for broth dilution antifungal susceptibility testing of yeasts; Proposed standard. NCCLS, publication M27-P, Villanova, Pa. NCCLS.
41. S. Khare, A. Trivedi, P. C. Kesavan and R. Prasad, *Int. J. Radiat. Biol.,* 1982, **42**, 369.
42. O. W. Griffith, *Anal. Biochem.,* 1980, **106**, 207.
43. J. K. Seydel, H. van der Goot and H. Timmerman, *Chemotherapy*, 1994, **40**, 124.
44. M. Geraghty, M. McCann, M. Devereux, F. Cronin, M. Curran and V. McKee, *Metal-Based Drugs*, 1999, **6**, 41.
45. D. J. Jamieson, D. W. S. Stephen and E. C. Terriere, *FEMS Microbiol. Letts.*, 1996, **138**, 83.
46. D. W. S. Stephen and D. J. Jamieson, *FEMS Microbiol. Letts.*, 1996, **141**, 207.
47. J. Bolard, *Biochim. Biophys. Acta*, 1986, **864**, 257, and references therein.
48. E. R. Stadtman, *Annun. Rev. Biochem.*, 1993, **62**, 797, and references therein.

**Received: September 11, 2000- Accepted: September 20, 2000 -**
**Received in revised camera-ready format: September 21, 2000**

